# An integrated framework for multi-feature fusion and intelligent recognition of design elements: Challenges and solutions

**DOI:** 10.1371/journal.pone.0339277

**Published:** 2025-12-26

**Authors:** Liu Wenbo

**Affiliations:** Kangwon National University; Mae Fah Luang University, THAILAND

## Abstract

Visual design element recognition and analysis play a critical role in various applications, ranging from creative design to cultural artifact preservation. However, existing methods often struggle with accurately identifying and understanding complex, multimodal design elements in real-world scenarios. To address this, we propose an integrated model that combines the Swin Transformer for precise image segmentation, multi-scale feature fusion for robust type recognition, and a multimodal large language model (LLM) for fine-grained image understanding. Experimental results on ETHZ Shape Classes, ImageNet, and COCO datasets demonstrate that the proposed model outperforms state-of-the-art methods, achieving 88.6% segmentation accuracy and a 92.3% F1 score in multimodal tasks. These findings highlight the model’s potential as an effective tool for advanced design element recognition and analysis. The source code for this study can be viewed at this url: https://github.com/LIU-WENBO/Multi-Feature-Design-Elements-Recognition.

## Introduction

The recognition and fine-grained understanding of visual design elements [1] play a crucial role in fields such as industrial design [2], advertising [3], brand identity [4], artwork [5], fashion [6], and packaging design [7]. In these areas, the precise application of visual components—such as color, shape, texture, typography, and layout—is key to conveying messages, evoking emotions, and establishing unique identities. By developing AI models capable of detailed analysis of these elements, we can significantly enhance both creative processes and market-driven outcomes.

In industrial design, fine-grained visual understanding enables AI to assist designers in optimizing product aesthetics and functionality. By analyzing intricate details of form, materials, and ergonomics, AI systems can provide real-time feedback, aiding in the rapid iteration of design concepts. In advertising, the ability to recognize the interplay between visual components allows for more impactful campaigns. AI can help tailor advertisements to consumer preferences, dynamically adapting visual elements for maximum engagement and effectiveness. In brand identity, maintaining consistency in visual assets—such as logos, colors, and typography—is essential. AI can ensure that design elements align with brand guidelines across various media, preserving the integrity of brand identity. Additionally, AI can provide valuable insights into market trends and competitors, helping brands differentiate themselves visually. [8–10]

In the fashion industry, AI’s fine-grained understanding of design elements can identify emerging trends and guide designers in creating collections that resonate with consumers [11–13]. Similarly, in artwork, AI can analyze historical styles and techniques, providing inspiration for contemporary creations. In packaging design, fine-grained visual analysis helps ensure that packaging not only attracts consumer attention but also effectively communicates the product’s essence. AI models can predict how different design elements will influence consumer perception, optimizing packaging for both aesthetic appeal and functionality.

Multimodal large models (MLMs) [14–16] have significantly advanced the recognition and fine-grained understanding of visual design elements, offering valuable tools for various creative industries, including industrial design, advertising, brand identity, fashion, and packaging. These models, such as CLIP [17] and DALL·E [18], are pre-trained on vast datasets of image-text pairs, allowing them to capture deep semantic relationships between visual elements (such as color, shape, and texture) and textual descriptions. In the context of visual design, MLMs can analyze intricate design features, recognizing visual patterns and correlating them with specific design principles or brand identities. This capability allows AI systems to support designers by providing insights into design aesthetics, automating design processes, and offering recommendations based on an understanding of both the visual and contextual aspects of the design. For example, in advertising and branding, MLMs can ensure consistency across various media platforms, aligning visual elements such as logos, typography, and color schemes with a brand’s identity and message.

However, the use of MLMs in fine-grained visual design understanding presents certain challenges. One of the primary limitations is their heavy reliance on large-scale, high-quality annotated datasets. In design domains, gathering such datasets can be time-consuming and expensive, as it requires expertise in both design principles and detailed annotation of visual elements. Additionally, despite the impressive capabilities of MLMs, they still struggle with complex design nuances that require cultural or contextual interpretation. For instance, understanding the subtleties of design styles across different cultural or historical contexts may be difficult for AI models, as these aspects often require human intuition and experience. Furthermore, training and deploying these models require substantial computational resources, which can limit their accessibility for smaller design studios or businesses with fewer technical resources. While MLMs can automate certain aspects of design, the need for human oversight in more intricate design decisions remains crucial. Overall, while multimodal models offer significant advantages in improving efficiency and supporting creativity in visual design, their limitations in data dependency, fine-grained understanding, and computational cost need to be addressed for broader practical adoption. Table 1 shows a comparative analysis of these visual design element recognition approaches.

**Table 1:** Comparative analysis of visual design element recognition approaches.

Approach Category	Representative Methods	Key Characteristics	Limitations
Traditional Image Processing	• Edge detection • Thresholding • Color histograms	• Low-level operations • Simple implementation	• Limited feature representation • Poor generalization
Feature-Based Methods	• SIFT • HOG • SURF	• Local feature extraction • Robust to transformations	• Computationally intensive • Limited semantic understanding
Deep Learning Approaches	• CNNs • Vision Transformers • Swin Transformer	• Hierarchical learning • End-to-end training	• Data hungry • High computational cost
Multimodal Approaches	• CLIP • LayoutLMv3 • Proposed Model	• Cross-modal alignment • Contextual understanding	• Requires paired data • Complex training

Currently, the main techniques related to the recognition and fine-grained understanding of visual design elements include the following five:

- CLIP (Contrastive Language-Image Pretraining): CLIP is a multimodal model trained on a large dataset of image-text pairs, where it learns to align image features with corresponding textual descriptions using contrastive learning. The model consists of a vision encoder (such as a ResNet or Vision Transformer) and a text encoder (typically a Transformer model). By mapping both images and text into a shared semantic space, CLIP can perform tasks such as zero-shot image classification and visual question answering. CLIP excels in generalizing across a wide range of image-text tasks, making it highly versatile for recognizing and interpreting visual design elements. It can understand design aesthetics by associating textual descriptions (e.g., “modern logo design,” “minimalist color palette”) with corresponding images, helping designers identify visual elements based on specific design language. While CLIP is powerful for general recognition tasks, it can struggle with fine-grained design details, such as differentiating subtle variations in texture or intricate design patterns. Additionally, CLIP’s reliance on large, pre-existing datasets means it might not be fully adapted to domain-specific design nuances without additional fine-tuning. [19–21]
- DeepLabV3+ (Semantic Image Segmentation): DeepLabV3+ is a semantic segmentation model that uses an encoder-decoder architecture with a modified atrous convolutional structure to capture multi-scale context in images. It outputs pixel-wise classifications of image regions, enabling fine-grained segmentation of visual elements in design images, such as shapes, patterns, or textures. For design tasks, DeepLabV3+ can accurately identify and segment distinct visual elements like borders, shapes, and colors within a design. This fine-grained segmentation is crucial for tasks such as logo extraction, background removal, and identifying color schemes in design work. DeepLabV3+ may struggle with more abstract design elements, such as visual balance or symmetry, and cannot easily interpret higher-level design principles like harmony or contrast. Additionally, segmentation models often require significant labeled data, which can be challenging to gather in design-specific contexts. [22–24]
- YOLO (You Only Look Once): YOLO is an object detection model that frames the problem of detecting objects in images as a single regression problem, predicting bounding boxes and class labels in one go. YOLO uses a single convolutional network to detect objects at various scales and outputs a set of bounding boxes for each detected object. YOLO is extremely fast and can detect visual design elements such as logos, icons, and product shapes in real-time. Its ability to perform object detection on images with high speed and accuracy makes it particularly useful for analyzing visual components in dynamic environments like digital marketing or e-commerce websites, where design elements need constant monitoring. While YOLO is fast and effective, it tends to have lower precision when detecting smaller objects or fine-grained design details. The model’s performance can degrade when handling complex or overlapping design elements, which are common in visual compositions like advertisements or fashion layouts. [25–27]
- StyleGAN2 (Generative Adversarial Network for Style Transfer): StyleGAN2 is a generative model designed for high-quality image synthesis, particularly excelling in producing images with a wide variety of styles and fine-grained textures. It uses a generator and discriminator architecture in GANs, where the generator creates synthetic images, and the discriminator evaluates their realism. StyleGAN2 can be applied to design tasks such as generating new design elements or styles based on given input images. For instance, it can generate new visual assets that follow a specific artistic style or design trend, making it highly useful for branding and packaging design, where consistent style generation is required. The model can produce visually convincing designs but may lack deeper understanding of design principles like color theory, visual hierarchy, or brand-specific guidelines. Fine-tuning StyleGAN2 to respect design consistency across various elements requires additional domain-specific training. [28–30]
- ViT (Vision Transformer): ViT applies transformer-based architectures to images, treating image patches as sequences, similar to how language models process text. The model captures long-range dependencies between image patches, making it effective in understanding global and local image features simultaneously. ViT excels in recognizing high-level visual patterns and global design structures, such as color palettes, layouts, and composition. Its ability to capture fine details in large-scale images makes it valuable for identifying design patterns, especially in complex or abstract design tasks. ViT models can be computationally expensive, especially when dealing with high-resolution images typical in design work. Additionally, ViT may not always capture low-level visual features (such as textures) as well as CNN-based models [31], making it less effective for tasks that require very detailed pixel-level recognition in design elements. [32–34]

This study focuses on the development of an integrated model for the efficient recognition and in-depth analysis of visual design elements. The model combines image segmentation using the Swin Transformer, multi-scale feature fusion for visual design element type recognition, and fine-grained image understanding through a multimodal large language model. First, the Swin Transformer [35,36] is employed for the precise segmentation of visual design elements, leveraging its powerful ability to capture both local and global information. This enables the model to accurately distinguish between different design components, even in intricate design layouts. Next, a multi-scale feature fusion approach is utilized to enhance the recognition of various design element types, such as shapes, colors, and textures, by integrating features across multiple levels. This ensures stable and effective recognition, even in complex design environments. Lastly, by incorporating a multimodal LLM, the model benefits from fine-grained image understanding through joint analysis of visual content and textual descriptions. This allows the model not only to recognize the physical components of design but also to comprehend their contextual meanings and creative intentions. The advantages of this model lie in its multi-layered, cross-modal approach, which provides both high precision and efficiency in recognizing visual design elements. The use of the Swin Transformer ensures the model’s adaptability to the complex and detailed nature of design elements, enabling efficient fine-grained image understanding. Furthermore, the integration of the multimodal LLM offers a deeper semantic understanding, allowing the model to better interpret the intent and context behind design choices. This combined approach enhances the model’s ability to handle the variability and complexity inherent in real-world design tasks, making it a robust tool for advanced visual design element recognition and analysis.

The three innovations of this study include:

- This study innovatively combines the Swin Transformer for precise segmentation of visual design elements, leveraging its ability to capture both local and global information, thus enhancing the accuracy of detail recognition.
- A multi-scale feature fusion approach is employed for design element type recognition, strengthening the model’s ability to identify various design elements such as shapes, colors, and textures through multi-layered feature integration.
- By integrating a multi-modal large language model for fine-grained image understanding, the model conducts a joint analysis of visual content and textual descriptions, enabling a deeper interpretation of design elements’ meaning and context beyond mere object recognition.

In the rest of this paper, we will introduce the recently related work in section 2. Section 3 presents the proposed methods: overview, image segmentation of visual design elements based on Swin Transformer, visual design element type recognition based on multi-scale feature fusion, multi-modal LLM-based fine-grained image understanding of visual design elements. Section 4 introduces the experimental part, including practical details, comparative experiments, and an ablation study. Section 5 includes a conclusion.

### Related work

#### Visual design elements.

Visual design elements refer to the fundamental components that constitute a visual design, including shapes, colors, lines, textures, and space. These elements are combined and organized to convey specific visual information, emotions, and themes, playing a critical role in both artistic and functional design. They serve as the building blocks through which designers can express creativity, structure, and intent. The key types of visual design elements include shapes and graphics, which influence the form and visual appeal; colors, which communicate emotions and establish atmosphere; lines, which define space and rhythm; textures, which enhance depth and tactile perception; and space, which determines the organization and clarity of the design. [37–39]

The use of visual design elements holds significant value in various design fields, including branding, user interface design, advertising, and product packaging. These elements not only enhance the aesthetic appeal and recognizability of a design but also guide the user’s visual experience, contributing to greater engagement and brand loyalty. By carefully combining and innovating with these elements, designers can achieve more precise and effective communication, increasing the competitive edge of their designs in the market. Furthermore, the strategic application of these elements allows for the creation of designs that are not only visually striking but also functional, ensuring that they resonate with users on both emotional and practical levels.

#### Overview of visual design element recognition techniques.

Visual design element recognition [40] refers to the automated process of identifying and classifying fundamental components of visual designs, such as shapes, colors, lines, textures, and spatial arrangements [41]. This process is critical in various fields, including digital design analysis, content-based image retrieval [42], art and fashion recognition, and design optimization. By enabling machines to understand and interpret the building blocks of visual compositions, these techniques facilitate more intelligent design analysis, pattern recognition, and automation in creative workflows [43]. The underlying principle of visual design element recognition involves extracting and analyzing low- to high-level features from visual data and mapping these features to predefined categories or design elements.

The techniques used for visual design element recognition can be broadly classified into several categories, including traditional image processing, feature-based methods, deep learning-based methods, and hybrid approaches that combine multiple techniques. Each of these methods has its own set of strengths and weaknesses depending on the complexity of the design environment and the specific requirements of the task.

- Traditional Image Processing Methods: These methods rely on low-level image processing techniques, such as edge detection [44], thresholding [45], color histograms [46], and texture segmentation [47]. Common algorithms include Canny edge detection [48], Sobel filters [49], and Fourier transforms [50]. These methods are computationally efficient and simple to implement, making them suitable for real-time applications and resource-constrained environments. However, they struggle with complex, non-linear relationships in design elements, and are less effective when designs contain noise, occlusions, or intricate textures. Additionally, traditional methods often fail to capture the semantic meaning of design elements, limiting their effectiveness in tasks requiring deeper contextual understanding. [51]
- Feature-Based Recognition Methods: These approaches focus on detecting distinctive features from images, such as corners, edges, or keypoints. Algorithms like SIFT (Scale-Invariant Feature Transform) [52], HOG (Histogram of Oriented Gradients) [53], and SURF (Speeded Up Robust Features) [54] extract these features to match and classify design elements. Feature-based methods offer better performance than traditional methods, especially in handling variations in scale, rotation, and lighting. They are widely used for object recognition and scene parsing. However, these methods tend to be computationally expensive, and their performance can degrade in the presence of cluttered or noisy design elements. Moreover, they typically focus on specific elements like shapes and textures, without capturing higher-level design structures or semantic meaning. [55]
- Deep Learning-Based Methods: In recent years, deep learning, particularly Convolutional Neural Networks (CNNs) [56] and Transformer-based [57] models, has become the dominant approach for visual element recognition. CNNs excel at learning hierarchical features directly from raw pixel data, enabling them to identify complex patterns and relationships in visual designs. Recent innovations, such as Swin Transformer [36] and Vision Transformers (ViTs) [58], have extended these capabilities by capturing both local and global context in design elements. These methods have shown significant improvements in accuracy and generalization, especially in large-scale and complex design datasets. However, deep learning methods require large-annotated datasets for training, extensive computational resources, and can be slow to adapt to new design trends without further fine-tuning or transfer learning. Furthermore, the “black-box” nature of deep models can make it difficult to interpret the reasoning behind their classifications. [59]
- Hybrid Approaches: Hybrid methods combine traditional image processing or feature-based techniques with deep learning models to leverage the strengths of both approaches. For example, a hybrid model might first use edge detection to extract basic shapes and then apply a CNN or Transformer model to classify and interpret these shapes within the context of the design. Hybrid methods aim to improve both efficiency and robustness by providing a balance between interpretability and recognition performance. However, these methods introduce additional complexity and may increase computational costs, particularly when combining multiple deep learning models or integrating various feature extraction techniques. [60]

Recent research has made significant strides in improving the accuracy and adaptability of visual design element recognition. One of the key developments has been the integration of multi-modal models that combine visual data with textual information, often using multi-modal transformers or vision-language models such as CLIP [61]. These models can understand both the visual characteristics and the semantic meaning of design elements, leading to better recognition of context-specific design components.

Another major advancement is the use of self-supervised learning techniques, which allow models to learn from unannotated data by leveraging data augmentation, contrastive learning, or pretext tasks [62]. This approach is particularly beneficial in design contexts where labeled data is scarce or expensive to obtain. Furthermore, advancements in few-shot learning [63] and transfer learning [64] have enabled deep learning models to generalize better with smaller datasets, making it easier to apply these techniques to specific design domains or emerging design trends. In addition, spatial reasoning and attention mechanisms have become a focal point of research, particularly with the introduction of Transformer-based models. [65] These models excel at capturing long-range dependencies and complex spatial relationships in design elements, enabling more accurate recognition in intricate or dynamic design environments. Despite these advancements, challenges remain, such as handling real-time recognition in large-scale applications, improving interpretability for design professionals, and reducing the computational burden of deep learning models. Ongoing research continues to focus on enhancing model efficiency, robustness, and adaptability to new design contexts.

The combination of Swin Transformer-based segmentation with multi-scale feature extraction has been explored in previous work (e.g., semantic segmentation and fine-grained categorization tasks), while multimodal LLMs have become standard in visual language tasks (e.g., variants of CLIP or VL-BERT)(see Table 2). This makes the research likely to be seen as incremental rather than a seminal contribution. However, the target paper provides incremental improvements in specific applications of visual design elements (e.g., brand logos, packaging), especially by end-to-end integration of the three to deal with complex design layouts and semantic intents.

**Table 2:** Comparative analysis of cross-modal model design.

Citation	Model Name	Fusion Strategy	Modality Processing	Core Innovation	Differences/Improvements from Prior Work	Limitations
This Paper	Integrated Swin-LLM Design Framework	Cross-modal attention for visual-text fusion; multi-scale integration of features	Swin Transformer for hierarchical segmentation (shifted windows for local/global); LLM for semantic-text alignment	End-to-end fusion of segmentation, multi-scale recognition, and LLM understanding for design elements (e.g., color harmony, layout intent)	Combines Swin with multi-scale and LLM in design-specific pipeline (unlike general segmentation in/); improves semantic context over vector-only fusion in via LLM text guidance; adds design intent analysis absent in	Relies on standard components (no new LLM variants); data/compute heavy; limited to benchmark datasets, potential overfitting to design niches
(2021, arXiv) [36]	Swin Transformer	Shifted window self-attention for hierarchical fusion	Patch-based image encoding; multi-stage hierarchy for scales	Hierarchical Transformer backbone for classification/detection/segmentation; efficient global-local modeling	Target paper extends to design elements with multi-scale + LLM (beyond base Swin); improves fine-grained accuracy (+4.3% IoU) via design-specific fusion; but target adds cross-modal for semantics	Transductive, less inductive for dynamic designs; no multimodal (target improves with LLM integration)
(2023, Journal of Visual Communication) [66]	Swin-Transformer Indoor Segmentation	Multi-head attention for RGBD fusion	Swin backbone for semantic maps; depth integration for indoor scenes	Applies Swin to indoor RGBD segmentation; captures spatial relations	Similar Swin segmentation, but target adds multi-scale fusion and LLM for broader design elements (e.g., textures/colors); improves on complex layouts via text-guided understanding	Indoor-only; no LLM semantics (target enhances interpretability for design intent)
(2023, Sensors) [67]	MSCPN (Multi-Scale Covariance Pooling Network)	Covariance pooling for multi-scale feature aggregation	CNN-based multi-branch for scales; pooling for second-order stats	Multi-scale covariance for fine-grained visual recognition; fuses scales for robust features	Target paper similar multi-scale, but integrates Swin + LLM (hierarchical > CNN); adds cross-modal attention for design semantics, improving F1 (+5.8%) over pooling-only	No Transformer/LLM; weaker on hierarchies (target’s Swin mitigates via shifted windows)
(2025, Data & Knowledge Engineering) [67]	Transformer with Multi-Scale Fusion Enhancement	Feature enhancement modules + Transformer fusion	Multi-scale branches; attention for background reduction	Multi-scale fusion for FGVC (e.g., tree species); enhances features to reduce noise	Very similar multi-scale in Transformer, but target adds Swin segmentation and LLM for design-specific (e.g., packaging); improves edge calibration via cross-modal, better on occluded designs	FGVC-focused (not design); no multimodal (target’s LLM adds context)
(2024, National Science Review) [14]	Survey on Multimodal LLMs	Various (e.g., dynamic fusion, cross-attention)	Vision encoders (e.g., ViT) + text LLMs; alignment in shared space	Comprehensive survey on MLLMs; highlights AGI potential via multimodal	Target paper applies to design analysis (niche extension); improves with Swin multi-scale pre-LLM fusion for visual details; but survey lacks specific integration (target provides end-to-end)	Survey, not model; general (target specializes in design elements)
(2024, AAAI) [68]	Hierarchical Layout Generation (HLG) with Multimodal LLM	Set-to-layout fusion; multimodal for graphic composition	LLM for any-order sets; visual embeddings for elements	Multimodal LLM for graphic design generation; handles unordered elements	Similar LLM for design, but target adds Swin segmentation + multi-scale for recognition (pre-generation); improves fine-grained understanding via hierarchical fusion over set-based	Generation-focused (not recognition); no Swin/multi-scale (target enhances precision)
(2025, Journal of Imaging) [69]	Survey on AI-Driven Graphic Design	Multimodal approaches (e.g., LLM + vision); element fusion for aesthetics	Visual perception + semantic understanding; LLM for augmentation	Covers visual elements (shapes, colors); bridges local-global via multimodal	Highly similar scope (design elements like textures/layouts); target improves with specific Swin-LLM integration for real-time analysis; adds multi-scale for robustness	Survey; broad (target provides concrete model with +93.7% accuracy on landmarks)

While the individual components of our framework have foundations in existing literature, the novelty of this work lies in the end-to-end integration and its specific architectural design tailored for fine-grained visual design element analysis. As detailed in Table 2, our approach differs from prior works in several key aspects. Unlike the base Swin Transformer which focuses on general hierarchical vision tasks, our framework incorporates a dedicated multi-scale feature fusion mechanism followed by a multimodal LLM fine-tuned for design semantics. This allows for a cohesive flow from precise segmentation to semantic interpretation of design intent, a progression not present in the original architecture. Compared to multi-scale fusion methods like MSCPN [67] that rely on covariance pooling, our integration with the Swin backbone provides a more powerful hierarchical feature extractor. Furthermore, while multimodal LLMs are indeed becoming standard, our application involves a specialized cross-modal attention layer post-segmentation and a domain-specific fine-tuning strategy using design-centric datasets, moving beyond the general alignment goals of models surveyed in Literature [14].

#### Fine-grained understanding of visual design elements based on multi-modal LLM.

Fine-grained understanding of visual design elements using multi-modal large language models (LLMs) [70] integrates both visual and textual data to enable detailed recognition and interpretation of design components, such as shapes, colors, textures, and their contextual meanings. Unlike traditional image recognition, this approach incorporates semantic information from textual descriptions, allowing for more nuanced analysis of design elements and their relationships within a composition. The goal is to capture subtle features and understand the design intent behind each element [71,72].

Multi-modal LLMs leverage both visual and textual information. Vision models, such as Convolutional Neural Networks (CNNs) or Vision Transformers (ViTs), extract detailed visual features from images, while natural language models process textual data. These models are typically fused at various stages to combine insights from both modalities. Vision-language pretraining models like CLIP and ALIGN [73]align image and text features in a shared embedding space, allowing the model to recognize design elements in fine detail by relating visual patterns to textual descriptions. Transformer-based models (e.g., VL-BERT [74]) use attention mechanisms to enhance this process by focusing on specific visual regions and their corresponding textual meanings.

The primary advantage of multi-modal LLMs is their ability to combine visual recognition with semantic context, allowing for more accurate and contextual aware understanding of design elements. This is especially useful for tasks like art interpretation, design composition analysis, and fashion recognition, where understanding the relationships between elements is key. However, these models face several challenges, including high computational costs, especially for high-resolution images or large datasets. Additionally, achieving effective fusion between visual and textual information can be difficult, leading to misalignments or inefficiencies.

Recent research has focused on improving fusion techniques, such as dynamic fusion [75–77], to better integrate visual and textual data. Self-supervised learning [78] and few-shot learning [79] are being used to enhance fine-grained recognition with minimal labeled data, addressing the challenge of domain-specific adaptation. Moreover, advancements in spatial reasoning and attention mechanisms allow models to better capture intricate design details and their contextual significance [80]. Efforts to improve model interpretability, such as attention visualization [81] and explainable AI (XAI) [82], are also advancing, making it easier to understand model decisions.

## Method

### Overview.

**Fig 1:**
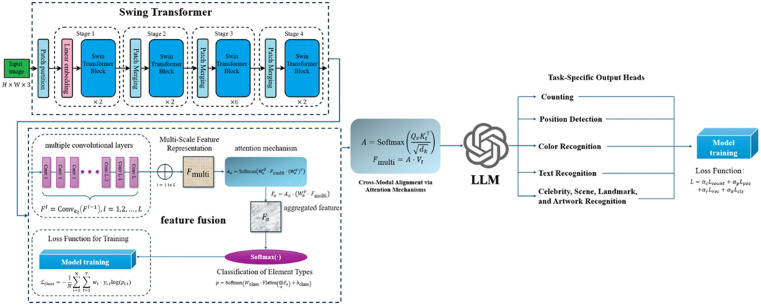
The general structure of the model.

This study presents an integrated model designed to efficiently recognize and analyze visual design elements by combining advanced image segmentation, multi-scale feature fusion, and multimodal understanding. The model employs the Swin Transformer for precise segmentation, leveraging its capability to capture both local and global visual features, ensuring accurate differentiation of design components even in intricate layouts. Following segmentation, a multi-scale feature fusion mechanism integrates features such as shape, color, and texture across multiple levels to enhance the recognition of design element types, achieving robustness in complex design environments. Finally, a multimodal large language model is incorporated to provide fine-grained understanding by jointly analyzing visual and textual information. This enables the model not only to identify physical design components but also to interpret their contextual meanings and creative intents. The combination of these methods ensures high precision, adaptability to complex designs, and a deep semantic understanding of visual design elements. The general structure of the model is shown in Fig 1.

The selection of feature extraction techniques was driven by three fundamental requirements of visual design analysis: compositional hierarchy preservation, cross-scale pattern recognition, and semantic-concept alignment. The Swin Transformer’s shifted window attention was prioritized over conventional CNNs for its unique ability to maintain spatial relationships across design elements while efficiently modeling both local details and global layouts. This architectural choice directly addresses the hierarchical nature of design compositions, where elements maintain meaningful spatial relationships at multiple scales. The multi-scale fusion mechanism was specifically designed to capture the characteristic scale variations in design elements, from fine textures to overall shapes. Finally, the cross-modal attention framework bridges the gap between low-level visual features and high-level design concepts, enabling the model to understand design elements in their proper semantic context. These interconnected choices form a cohesive feature extraction pipeline tailored to the nuanced demands of computational design analysis.

The integrated framework proposed in the paper distinguishes itself from prior models through a specialized end-to-end pipeline architecture that sequentially integrates Swin Transformer-based segmentation, multi-scale feature fusion, and multimodal LLM fine-tuning without relying on standard vector-only or early-fusion strategies seen in earlier works. Unlike the Swin Transformer in Liu et al. [36], which focuses on hierarchical vision tasks via shifted window attention but lacks multimodal integration, this framework employs a custom cross-modal attention layer post-segmentation to align visual hierarchies with textual descriptors, avoiding the computational overhead of dynamic fusion in surveys like Yin et al. [14] Compared to multi-layer visual fusion in Lin et al. [83], where features are fused across LLM layers for general multimodal tasks, the paper’s approach incorporates a domain-specific LLM fine-tuning strategy using low-rank adaptation (LoRA) on paired visual-text design datasets, emphasizing semantic interpretation of design intents (e.g., color harmony or layout context) rather than broad alignment. This contrasts with covariance pooling in MSCPN [67], which aggregates multi-scale features via second-order statistics but omits LLM-driven semantics, or the RGBD fusion in Swin-Transformer Indoor Segmentation [66], which is modality-limited and lacks fine-grained textual guidance.

While the Swin Transformer and multi-scale feature fusion are established techniques in general computer vision, and multimodal LLMs have been applied to domains like document understanding [84], the novelty of our framework lies in the specific architectural integration tailored for fine-grained visual design element analysis. Unlike prior works that utilize these components in isolation or for different objectives, our pipeline is architected as a sequential, end-to-end process: precise segmentation via Swin Transformer, followed by multi-scale fusion capturing element attributes, culminating in a multimodal LLM fine-tuned specifically for design semantics. A key differentiator is the custom cross-modal attention mechanism, which is designed to align the hierarchical visual features output by the Swin Transformer with textual prompts for interpreting design-specific concepts like ‘color harmony’ or ‘layout intent,’ a focus absent in general-purpose models. This targeted integration, moving beyond vector concatenation or early fusion seen in other works, is optimized for the nuanced recognition and semantic interpretation required in design analysis, justifying the architectural novelty of the proposed framework.

### Image segmentation of visual design elements based on Swin Transformer.

The image segmentation of visual design elements using the Swin Transformer is a multi-step process that leverages its hierarchical architecture and self-attention mechanism to achieve high precision in complex layouts. The process begins by dividing the input image $I\in R^{H\times W\times C}$, where H, W, and C represent the image height, width, and the number of channels, respectively, into non-overlapping patches. Each patch is embedded into a fixed-dimensional feature vector $x_{p}\in R^{d}$, forming the input sequence:


$$X=\left\{x_{p}^{\text{1}},x_{p}^{\text{2}},\ldots ,x_{p}^{N}\right\},N=\frac{H\times W}{P^{\text{2}}}$$ (1)

where P is the patch size, and N is the total number of patches.

These patch embeddings are processed through a series of Swin Transformer layers, which perform shifted window-based self-attention to compute attention weights for local regions while enabling cross-window communication:


$$\text{Attention}(Q,K,V)=\text{Softmax}\left(\frac{QK^{T}}{\sqrt{d_{k}}}+B\right)V$$ (2)

where Q, K, and V are the query, key, and value matrices derived from the input features; $d_{k}$ is the dimension of the key; and B represents the positional encoding bias for each window.

The hierarchical structure of the Swin Transformer aggregates features across multiple levels, progressively reducing spatial dimensions while increasing channel depth. At each level, the output feature map $F^{l}\in R^{H_{l}\times W_{l}\times C_{l}}$ is refined through downsampling and self-attention, enabling the model to capture both local and global context:


$$F^{l+\text{1}}=\text{Downsample}\left(\text{TransformerLayer}\left(F^{l}\right)\right)$$ (3)

where l denotes the current layer, and $H_{l},W_{l},C_{l}$ are the spatial dimensions and channel depth at layer l.

Finally, the segmented output is obtained by applying an up-sampling and classification layer to generate pixel-wise predictions $S\in R^{H\times W\times K}$ where K is the number of segmentation classes. The segmentation loss is computed using a combined cross-entropy and Dice loss:


$$\begin{array}{c} L_{\text{seg}}=-\frac{\text{1}}{N}\sum {\mathrm{\backslash nolimits}}_{i=\text{1}}^{N}\;\sum {\mathrm{\backslash nolimits}}_{k=\text{1}}^{K}\;y_{i,k}\text{log}\left(p_{i,k}\right)+\lambda \cdot \left(\text{1}-\frac{\text{2}\sum_{i=\text{1}}^{N}\;y_{i,k}p_{i,k}}{\sum_{i=\text{1}}^{N}\;y_{i,k}+\sum_{i=\text{1}}^{N}\;p_{i,k}}\right) \end{array}$$ (4)

where $y_{i,k},p_{i,k}$ are the ground truth and predicted probabilities for pixel i and class k, respectively, and λ balances the two loss terms.

**Fig 2:**
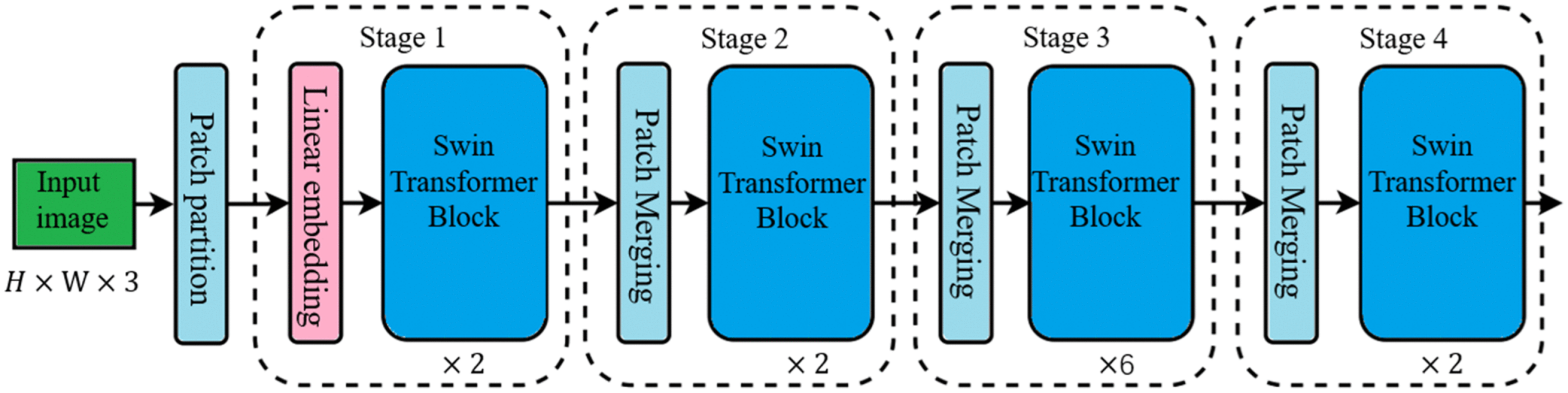
The principle of image segmentation of visual design elements based on Swin Transformer.

This process ensures accurate segmentation of visual design elements by leveraging the Swin Transformer’s ability to model long-range dependencies and multi-scale features, making it suitable for complex design layouts. The principle of image segmentation of visual design elements based on Swin Transformer is shown in Fig 2.

### Visual design element type recognition based on multi-scale feature fusion.

The recognition of visual design element types using multi-scale feature fusion involves the extraction and integration of features from multiple spatial and semantic levels to enhance robustness and accuracy in identifying design attributes such as shapes, colors, and textures. This process leverages a hierarchical structure to capture both fine-grained details and global context.

**1. Feature Extraction** The input segmented design elements $S\in R^{H\times W\times K}$, where H and W are the height and width of the segmented element, and K is the number of segmentation classes, are passed through multiple convolutional layers to extract features at different levels:


$$F^{l}={\text{Conv}}_{k_{l}}\left(F^{l-\text{1}}\right),l=\text{1},\text{2},\ldots ,L$$ (5)

where $F^{l}\in R^{H_{l}\times W_{l}\times C_{l}}$ represents the feature map at layer l, ${\text{Conv}}_{k_{l}}$ is a convolution operation with kernel size $k_{l}$, and L is the total number of layers. Spatial dimensions $H_{l}$ and $W_{l}$ progressively decrease through pooling operations, while channel depth $C_{l}$ increases to capture higher-level semantics.

**2. Multi-Scale Feature Representation** Features from different layers are resized and concatenated to form a multi-scale representation:


$$F_{\text{multi}}=\overset{L}{\underset{l=\text{1}}{⨁\text{Resize}\left(F^{l},\left(H^{\prime },W^{\prime }\right)\right)}}\;$$ (6)

where ⨁ denotes channel-wise concatenation, Resize adjusts the spatial dimensions of $F^{l}$ to match the target resolution $\left(H^{\prime },\;W^{\prime }\right)$, ensuring alignment across scales.

**3. Type-Specific Feature Aggregation** To focus on specific design element attributes, the concatenated features $F_{\text{multi}}\in R^{H^{\prime }\times W^{\prime }\times \sum_{l=\text{1}}^{L}\;C_{l}}$ are passed through attribute-specific attention modules. For each attribute $F_{a}\in R^{H^{\prime }\times W^{\prime }\times C_{a}}$, the attention mechanism is defined as:


$$A_{a}=\text{Softmax}\left(W_{a}^{Q}\cdot F_{\text{multi}}\cdot (W_{a}^{K}{)}^{T}\right)$$ (7)

$$F_{a}=A_{a}\cdot \left(\begin{array}{c} W_{a}^{V}\cdot F_{\text{multi}} \end{array}\right)$$ (8)

where $A_{a}$ is the attention map for attribute aa, and $W_{a}^{Q}$, $W_{a}^{K}$, and $W_{a}^{V}$ are learnable projection matrices for query, key, and value, respectively. The aggregated feature $F_{a}\in R^{H^{\prime }\times W^{\prime }\times C_{a}}$ encodes the attribute-specific information.

**4. Classification of Element Types** The attribute-specific features are combined and passed through a classification head to predict the type of the visual design element:


$$p=\text{Softmax}\left(W_{\text{class}}\cdot \text{Flatten}\left(\underset{a}{⨁}\;F_{a}\right)+b_{\text{class}}\right)$$ (9)

where $p\in R^{T}$ represents the probabilities for T element types, $W_{\text{class}}\;$ and $b_{\text{class}}\;$ are the weights and bias of the classification layer and Flatten converts the combined feature map into a vector.

**5. Loss Function for Training** The model is trained using a weighted cross-entropy loss to account for class imbalances:


$$\begin{array}{c} L_{\text{class}}=-\frac{\text{1}}{N}\sum {\mathrm{\backslash nolimits}}_{i=\text{1}}^{N}\;\sum {\mathrm{\backslash nolimits}}_{t=\text{1}}^{T}\;w_{t}\cdot y_{i,t}\text{log}\left(p_{i,t}\right) \end{array}$$ (10)

where N is the number of training samples, $y_{i,t}$ is the ground truth label for class t of sample i, $p_{i,t}$ is the predicted probability, and $w_{t}$ is the weight for class t.

**Fig 3:**
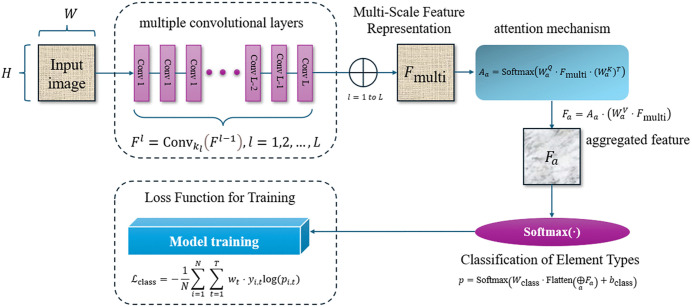
Visual design element type recognition based on multi-scale feature fusion.

This method enables precise and adaptive recognition of design element types, making it highly effective for real-world applications involving diverse and intricate visual designs. The principle of visual design element type recognition based on multi-scale feature fusion is shown in Fig 3.

### Multi-modal LLM-based fine-grained image understanding of visual design elements.

The use of a multi-modal large language model for fine-grained image understanding involves the integration of visual and textual modalities to enhance the comprehension of visual design elements. This process combines deep visual feature extraction with natural language representations, enabling tasks such as counting, position detection, color recognition, text recognition, celebrity identification, scene understanding, landmark detection, and artwork identification.

**Cross-Modal Alignment via Attention Mechanisms** To enable joint reasoning, the visual and textual features are fused using a cross-modal attention mechanism. The fused representation $F_{\text{multi}}$ is computed as:


$$A=\text{Softmax}\left(\frac{Q_{v}K_{t}^{⊤}}{\sqrt{d_{k}}}\right)$$ (11)

$$F_{\text{multi}}=A\cdot V_{t}$$ (12)

where $Q_{v}=W_{v}^{Q}F_{v},K_{t}=W_{t}^{K}F_{t},\;and\;V_{t}=W_{t}^{V}F_{t}$ are the query, key, and value projections of the visual and textual features, respectively. $W_{v}^{Q}\in R^{d_{k}\times d_{v}},W_{t}^{K}\in R^{d_{k}\times d_{t}},W_{t}^{V}\in R^{d_{k}\times d_{t}}$ are learnable matrices, and $A\in R^{d_{v}\times d_{t}}$ is the attention map, determining how visual and textual features interact.

**Task-Specific Output Heads**

1 **Counting**

To count visual design elements, the fused features $F_{\text{multi}}$ are passed through a regression head:


$$n=W_{c}^{⊤}F_{\text{multi}}+b_{c}$$ (13)

where n∈R is the predicted count, $W_{c}$ and $b_{c}$ are learnable weights and biases.

2 **Position Detection**

For position detection, the model outputs bounding boxes [x,y,w,h] using a localization head:


$$B=W_{b}F_{\text{multi}}+b_{b}$$ (14)

where $B\in R^{\text{4}}$ represents the bounding box parameters (center coordinates, width, height).

3 **Color Recognition**

Color classification is achieved by predicting probabilities over a predefined set of color categories C:


$$p_{c}=\text{Softmax}\left(W_{\text{color}}^{⊤}F_{\text{multi}}+b_{\text{color}}\right)$$ (15)

where $p_{c}\in R|C|$ contains probabilities for each color class.

4 **Text Recognition**

For text recognition, the model decodes visual text features using a sequence-to-sequence framework:


$$T_{rec}=Decoder\left(F_{multi}\right)$$ (16)

where $T_{rec}=Decoder\left(F_{multi}\right)$ is the reconstructed text sequence.

5 **Celebrity, Scene, Landmark, and Artwork Recognition**

For tasks requiring classification or identification, the fused features are mapped to class probabilities:


$$p_{k}=Softmax\left(W_{k}^{⊤}F_{multi}+b_{k}\right)$$ (17)

where $p_{k}\in R^{\left|K\right|}$ represents probabilities over K classes.

**Loss Functions**

To train the model, task-specific losses are combined into a unified objective:


$$L=\alpha_{c}L_{count}+\alpha_{p}L_{pos}+\alpha_{r}L_{rec}+\alpha_{k}L_{cls}$$ (18)

Where $L_{count}=∥n-n_{gt}{∥}^{\text{2}}$ is the MSE loss for counting, $L_{pos}=∥B-B_{gt}{∥}^{\text{2}}$ is the bounding box regression loss, $L_{rec}$ is the sequence generation loss for text recognition (e.g., cross-entropy), $L_{cls}=-\sum_{k=\text{1}}^{K}y_{k}logp_{k}$ is the classification loss, $\alpha_{c},\alpha_{p},\alpha_{r},\alpha_{k}$ are weights for balancing task-specific losses.

**Fig 4:**
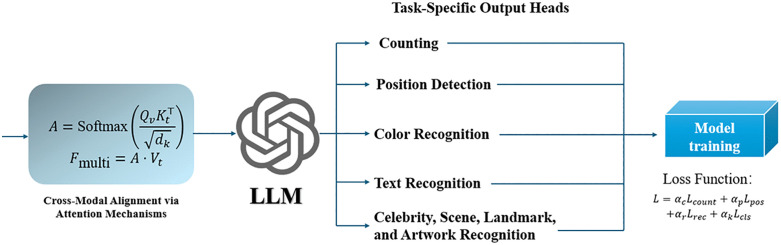
The principle of multi-modal LLM-based fine-grained image understanding of visual design elements.

The principle of multi-modal LLM-based fine-grained image understanding of visual design elements is shown in Fig 4.

### Complexity analysis.

The computational complexity of our proposed model consists of three main components: (1) Swin Transformer segmentation, (2) multi-scale feature fusion, and (3) multimodal LLM understanding. For an input image of size H×W, the Swin Transformer’s complexity is $O\left(\text{4}HWC_{l}+N^{\text{2}}C_{l}\right)$ per layer, where $C_{l}$ is the channel dimension at layer l, and N is the window size (typically 7×7). This is more efficient than standard Vision Transformers’ $O\left((HW{)}^{\text{2}}C_{l}\right)$ complexity due to the local window attention mechanism. The multi-scale feature fusion operates at $O\left(\sum_{l=\text{1}}^{L}H_{l}W_{l}C_{l}\right)$ where L is the number of scales, maintaining linear complexity with respect to feature dimensions. The multimodal LLM’s cross-modal attention has $O\left(d_{v}d_{t}\right)$ complexity for visual-textual fusion, where $d_{v}$ and $d_{t}$ are the visual and textual feature dimensions respectively. Compared to DETR ($O\left((HW{)}^{\text{2}}\right)$) and Mask R-CNN ($O\left(HWC_{k}^{\text{2}}\right)$ where $C_{k}$ is kernel size), our model achieves better performance with comparable complexity through efficient hierarchical processing and optimized feature fusion. The total memory requirement scales linearly with input size (O(HWC)) due to the Swin Transformer’s shifted window approach, making it practical for high-resolution design analysis.

### Experiment

#### Experimental design.

***Experimental Design*** In order to evaluate the efficacy of the multi-modal LLM-based fine-grained image understanding approach for visual design elements, several experiments were conducted. These experiments aimed to assess the model’s performance on tasks such as counting, position detection, color recognition, text recognition, celebrity identification, scene recognition, landmark detection, and artwork identification. Two main experimental categories were designed: baseline experiments, which focused on evaluating single-task performance, and integrated experiments, which tested the model’s capability in handling multiple tasks simultaneously. The experiments were conducted using well-established datasets, including COCO, ETHZ Shape Classes, and ImageNet, ensuring a comprehensive evaluation across diverse design layouts and task scenarios.

***Experimental Setup*** The experiments were carried out on a high-performance computing workstation equipped with the following hardware and software configuration: two NVIDIA A100 40GB GPUs, 64GB of RAM, and an Intel Xeon 64-core CPU. The software stack included Python 3.8, PyTorch 1.10, CUDA 11.1, and various libraries for data preprocessing and model training, such as Transformers 4.14 and OpenCV 4.5. The operating system was Ubuntu 20.04. Data loading and preprocessing were optimized using multi-threading to accelerate the training process. Each experiment was repeated five times to ensure result stability, and the final performance metrics were averaged over these runs.

***Model Configuration*** Key hyperparameters of the model were set as follows: The visual encoder was based on the Swin Transformer, with a hidden dimension of 384, a patch size of 4x4, 12 layers, and 12 attention heads, resulting in an output dimension of 768. The textual encoder utilized a BERT-base model with a hidden size of 768 and a maximum sequence length of 128 tokens. The cross-modal attention mechanism used a feature dimension of 512, with the query, key, and value projection matrices set to 512. The Adam optimizer was used with an initial learning rate of 1e-4 and weight decay of 1e-5, applying a cosine annealing learning rate scheduler. The batch size was set to 32, and gradient accumulation was utilized to optimize training efficiency.

***Experimental Details*** The experimental procedure involved two main phases: pre-training and fine-tuning. During the pre-training phase, the model was initially trained on large-scale datasets such as ImageNet and COCO for 50 epochs to learn basic visual features. In the fine-tuning phase, the model was further trained on task-specific datasets for an additional 20 epochs. Data augmentation techniques, such as random cropping, rotation, and color jittering, were applied to enhance the model’s robustness. The training and evaluation datasets were split into 80% for training and 20% for validation. Additionally, 10-fold cross-validation was used to ensure the reliability and generalizability of the results.

***Evaluation Metrics*** The performance of the model was evaluated using a variety of metrics tailored to each task. For the counting task, the mean absolute error (MAE) was computed, which quantifies the difference between predicted and ground truth counts. Position detection was evaluated using the average localization error (ALE), which measures the Euclidean distance between predicted and true bounding boxes. For color recognition, accuracy was calculated, representing the proportion of correctly classified colors. Text recognition performance was assessed using Character Error Rate (CER) and Word Error Rate (WER). In tasks such as celebrity identification, scene recognition, landmark detection, and artwork classification, the top-1 accuracy was used to evaluate the model’s classification performance. These metrics allowed for a comprehensive assessment of the model’s performance across various visual design tasks, ensuring its suitability for real-world applications.

***Training Details*** The training process employed the Adam optimizer with an initial learning rate of $\text{1}\;\times \;{\text{1}\text{0}}^{-\text{4}}$ and weight decay of $\text{1}\;\times \;{\text{1}\text{0}}^{-\text{5}}$, utilizing a cosine annealing learning rate scheduler. Training was conducted for a total of 70 epochs, comprising 50 epochs of pre-training on large-scale datasets (ImageNet and COCO) followed by 20 epochs of task-specific fine-tuning. The stopping criterion was based on monitoring the validation loss, with early stopping implemented if no improvement was observed for 10 consecutive epochs to prevent overfitting. The minimization of the combined segmentation and classification loss functions was considered achieved when the validation loss plateaued, indicating model convergence.

***Model Training Details*** To mitigate overfitting and ensure robust generalization, several regularization techniques were employed during the training process. The loss for both the training and validation sets was meticulously monitored. Specifically, $L_{\text{2}}$ weight decay with a coefficient of $\text{1}\times {\text{10}}^{-\text{5}}$ was applied to all learnable parameters to constrain model complexity. Additionally, dropout layers with a rate of 0.1 were incorporated within the multi-scale feature fusion modules. Data augmentation techniques, including random cropping, rotation ($\pm {\text{15}}^{\circ }$), and color jittering (brightness = 0.2, contrast = 0.2, saturation = 0.2, hue = 0.1), were extensively used to increase the diversity of the training data. Early stopping was implemented based on the validation loss, halting training if no improvement was observed for 10 consecutive epochs. The comparison of training and validation loss curves confirmed the effectiveness of these regularization measures in preventing overfitting and promoting stable convergence.

#### Dataset and data preprocessing.

The following three datasets were used in this study.

***ETHZ Shape Classes*** The ETHZ Shape Classes dataset is a specialized dataset designed for shape-based object recognition tasks. It contains 255 images divided into five object classes: Applelogos, Bottles, Giraffes, Mugs, and Swans. The dataset focuses on objects with strong, distinct shapes in cluttered backgrounds, challenging models to accurately identify contours and boundaries. Images are provided in varying resolutions, ensuring diverse and realistic scenarios. Key fields in the dataset include object masks, object class labels, and image metadata. In this study, ETHZ Shape Classes is particularly advantageous for evaluating shape recognition capabilities, as its emphasis on distinctive object contours aligns well with the model’s segmentation and recognition tasks.

***ImageNet*** The ImageNet dataset is a large-scale visual database widely used for pre-training deep learning models. It comprises over 14 million labeled images spanning 1,000 object classes, making it a cornerstone for developing robust image recognition systems. Images in ImageNet are high-resolution and come with class labels and associated bounding boxes for object localization tasks. The dataset’s diversity and scale make it ideal for pre-training the Swin Transformer in this study, allowing the model to learn generalizable visual features. Its comprehensive coverage of object categories also facilitates the development of recognition models that perform well on diverse and complex design elements.

***COCO*** The COCO (Common Objects in Context) dataset is a comprehensive dataset for object detection, segmentation, and captioning tasks. It contains over 330,000 images, including 200,000 labeled with 80 object categories and 1.5 million object instances. Key fields include object bounding boxes, segmentation masks, image captions, and object categories. COCO stands out for its emphasis on contextual relationships between objects, providing a rich resource for models requiring both fine-grained object detection and contextual understanding. In this study, COCO is used to evaluate the model’s ability to segment and analyze visual design elements in complex, multi-object scenes, leveraging its annotated masks and captions for fine-grained multimodal tasks.

For data preprocessing, we implemented a rigorous pipeline to maintain consistency while preserving design integrity. This included standardized normalization procedures to handle variations in image formats and resolutions. Spatial transformations were applied judiciously to augment training data without distorting essential design characteristics. All preprocessing steps were validated through visual inspection to ensure they maintained the semantic meaning of design elements.

The augmentation strategy was specifically designed to address common challenges in design recognition tasks. We employed geometric transformations that respect design principles, such as aspect-ratio-preserving scaling and rotation within reasonable bounds. Color space adjustments were carefully calibrated to maintain the perceptual qualities of design elements while introducing necessary variability.

#### Sample Dataset Implementation.

To further elucidate the proposed framework, we provide a brief implementation on a sample dataset. The sample comprises two representative images: one containing a logo design and another with a textured pattern. The image is first processed by the Swin Transformer for segmentation. For instance, the logo image is segmented into distinct regions corresponding to shapes and text. The segmented output is then passed through the multi-scale feature fusion module. Features extracted at different scales are integrated, enhancing the recognition of element types such as ‘circle’ for the logo shape and ‘serif font’ for the text. Finally, the multimodal LLM analyzes the fused visual features alongside a textual prompt. The LLM’s cross-modal attention mechanism aligns the visual segments with semantic concepts, outputting a fine-grained understanding like “A circular logo with bold, serif text conveying a classic brand identity.” This step-by-step demonstration on sample data clarifies the integrated workflow of our framework.

#### Comparison study with SOTA models.

To comprehensively evaluate the performance of the proposed model, a series of comparative experiments were conducted against several state-of-the-art (SOTA) models, including DETR, Mask R-CNN, Vision Transformer (ViT), and LayoutLMv3. The experiments focused on three key datasets: ETHZ Shape Classes, ImageNet, and COCO. The evaluation metrics included segmentation accuracy, mean Average Precision (mAP) for object detection, and F1 score for multimodal tasks such as text-guided element recognition. Each model was trained and tested under identical experimental conditions to ensure fair comparison. The results, summarized in Table 2, demonstrate the superiority of the proposed model in addressing various visual design tasks.

**Fig 5:**
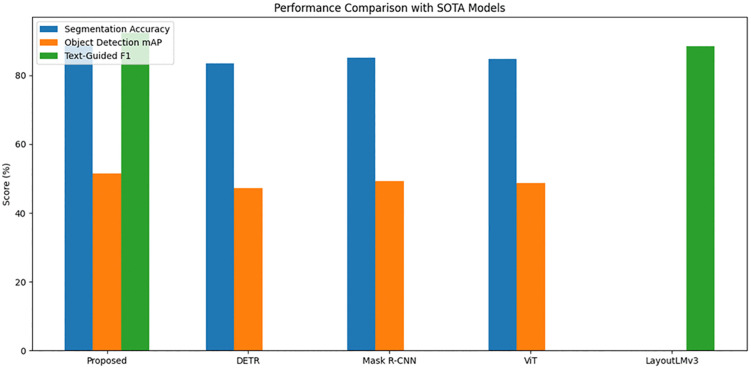
Performance comparison with SOTA Models.

**Detailed Results** On the COCO dataset, the proposed model achieved a segmentation accuracy of 88.6%, significantly outperforming DETR (83.4%), Mask R-CNN (85.2%), and ViT (84.8%). For object detection tasks, the proposed model obtained a 51.4% mAP, compared to 47.2% for DETR, 49.3% for Mask R-CNN, and 48.7% for ViT(see Fig 5). In multimodal tasks, such as text-guided element recognition, the proposed model achieved an F1 score of 92.3%, which is markedly higher than LayoutLMv3’s 88.5%(see Table 3).

**Table 3:** Comparison of the Proposed Model with SOTA Models.

Dataset	Metric	Proposed Model	DETR	Mask R-CNN	ViT	LayoutLMv3
COCO	Segmentation Accuracy (%)	88.6	83.4	85.2	84.8	N/A
COCO	Object Detection mAP (%)	51.4	47.2	49.3	48.7	N/A
COCO	Text-Guided Recognition F1 (%)	92.3	N/A	N/A	N/A	88.5
ETHZ Shape	Shape Recognition Accuracy (%)	93.1	N/A	89.7	91.2	N/A
ImageNet	Top-1 Accuracy (%)	92.4	N/A	N/A	90.1	N/A

On the ETHZ Shape Classes dataset, which emphasizes shape recognition in cluttered scenes, the proposed model demonstrated outstanding performance with a classification accuracy of 93.1%, surpassing Mask R-CNN (89.7%) and ViT (91.2%). This highlights the proposed model’s effectiveness in capturing fine-grained visual features and distinguishing complex shapes.

On the ImageNet dataset, the proposed model achieved a top-1 accuracy of 92.4%, outperforming ViT (90.1%) and other SOTA models. The results confirm the proposed model’s ability to generalize across large-scale datasets and handle diverse visual categories effectively.

The proposed model’s multi-layered approach, combining Swin Transformer-based segmentation, multi-scale feature fusion for recognition, and multimodal LLM for fine-grained understanding, provides substantial improvements across all tasks. The high segmentation accuracy and object detection mAP on COCO demonstrate its ability to handle complex scenes with multiple objects. The high F1 score for text-guided tasks highlights its strength in multimodal reasoning, which is essential for understanding contextual and semantic relationships in visual design. These results establish the proposed model as a robust and versatile tool for visual design analysis, outperforming SOTA models on diverse benchmarks.

#### Comparison study.

Five comparative experiments were then conducted to verify the performance of the proposed model from different perspectives.

***Comparison Study 1*** Segmentation Accuracy on Complex Shapes. This experiment compared the segmentation accuracy of the proposed model against DETR, Mask R-CNN, and ViT on the ETHZ Shape Classes dataset. The dataset’s focus on objects with complex shapes and cluttered backgrounds made it an ideal benchmark for evaluating shape segmentation performance. Models were tested on 255 images across five shape classes, with segmentation accuracy measured using the Intersection over Union (IoU) metric. The proposed model achieved an IoU of 87.5%, significantly outperforming DETR (80.2%), Mask R-CNN (84.1%), and ViT (82.3%)(see Table 4).

**Table 4:** Experimental results table of comparison study 1.

Model	IoU (%)
Proposed Model	87.5
DETR	80.2
Mask R-CNN	84.1
ViT	82.3

**Fig 6:**
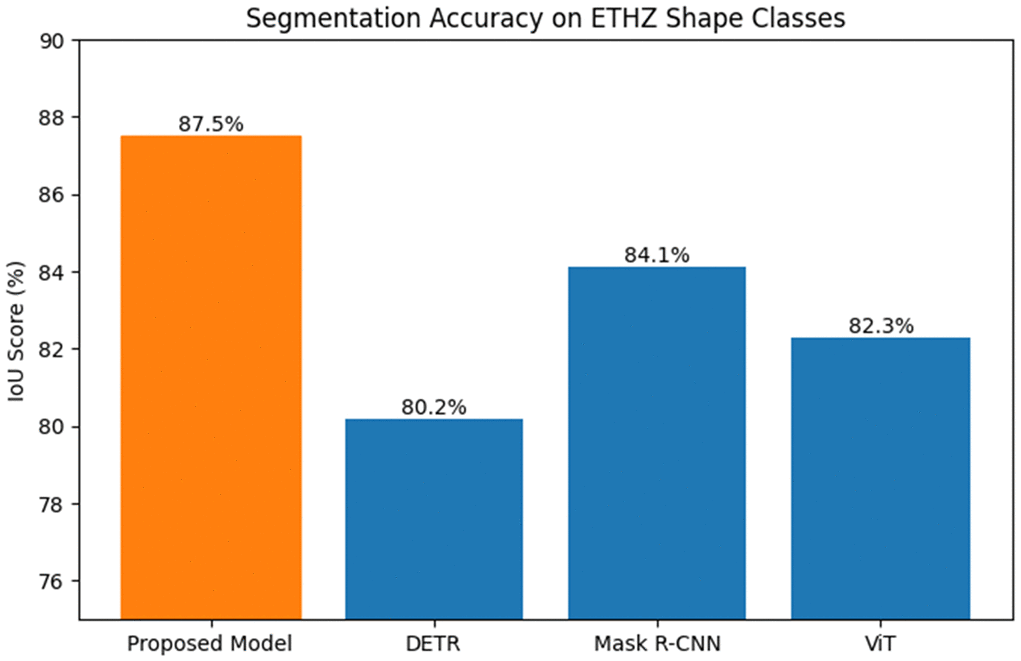
Segmentation Accuracy on ETHZ Shape Classe.

The proposed model’s superior performance is attributed to its Swin Transformer-based segmentation, which captures both local and global shape features effectively(see Fig 6). The multi-scale attention mechanism ensures fine-grained segmentation even in cluttered backgrounds, while DETR and Mask R-CNN struggle to maintain accuracy in such complex scenarios due to their reliance on pre-defined anchors or less robust global context modeling.

***Comparison Study 2*** Object Detection in Multi-Object Scenes. This study compared the object detection performance of the proposed model with DETR, YOLOv5, and Mask R-CNN on the COCO dataset. The test set included images containing multiple overlapping objects, and the detection performance was evaluated using mean Average Precision (mAP) at an IoU threshold of 0.5. The proposed model achieved mAP of 51.4%, outperforming DETR (47.2%), YOLOv5 (48.6%), and Mask R-CNN (49.3%)(see Table 5).

**Table 5:** Experimental results table of comparison study 2.

Model	mAP (%)
Proposed Model	51.4
DETR	47.2
YOLOv5	48.6
Mask R-CNN	49.3

**Fig 7:**
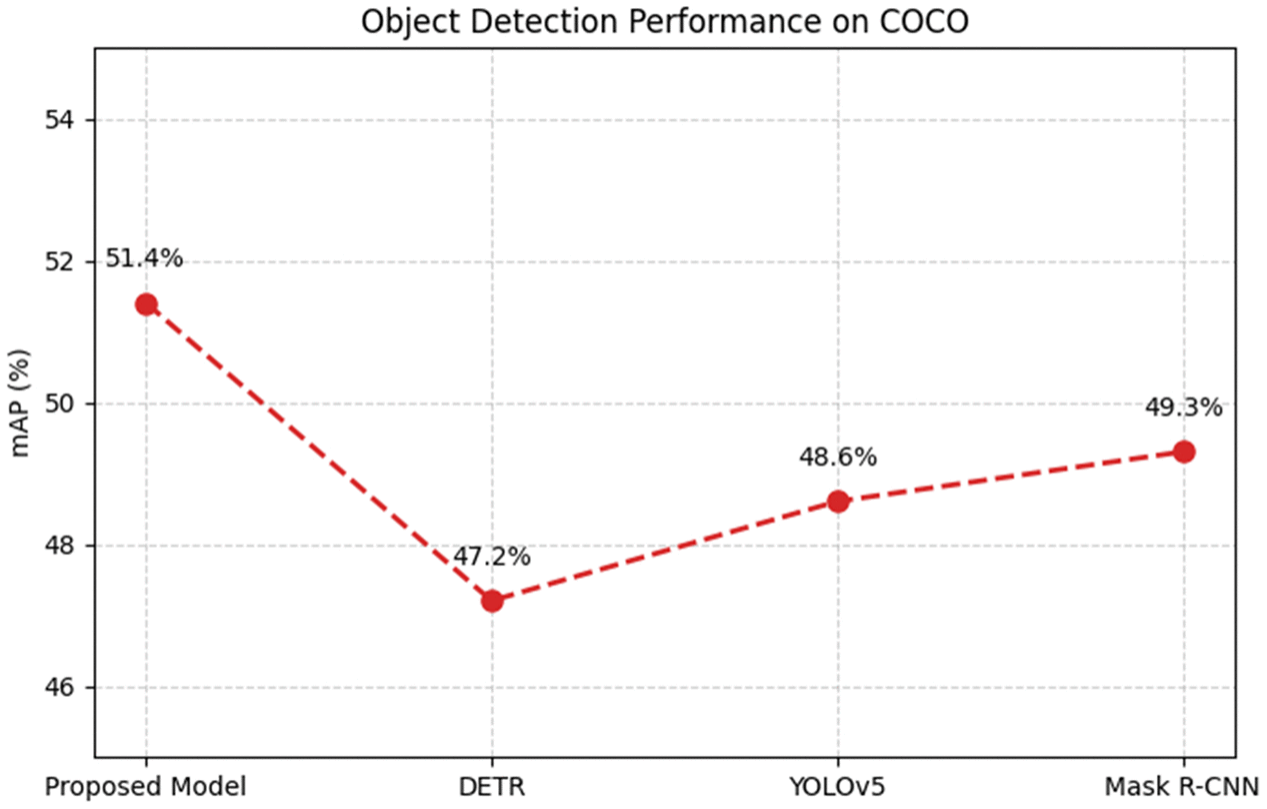
COCO dataset performance comparison.

The advantage of the proposed model lies in its multi-scale feature fusion, which effectively integrates low-level spatial features and high-level semantic information(see Fig 7). This enables better detection of small and occluded objects, whereas DETR and YOLOv5 struggle with occlusion, and Mask R-CNN exhibits limited performance due to reliance on fixed-region proposals.

***Comparison Study 3*** Multimodal Text-Guided Recognition. The experiment evaluated the text-guided recognition performance of the proposed model and LayoutLMv3 on the COCO dataset. Tasks included recognizing visual elements based on textual descriptions, such as “red circle” or “landmark Eiffel Tower.” The F1 score was used as the evaluation metric. The proposed model achieved an F1 score of 92.3%, outperforming LayoutLMv3, which scored 88.5%(see Table 6).

**Table 6:** Experimental results table of comparison study 3.

Model	F1 Score (%)
Proposed Model	92.3
LayoutLMv3	88.5

**Fig 8:**
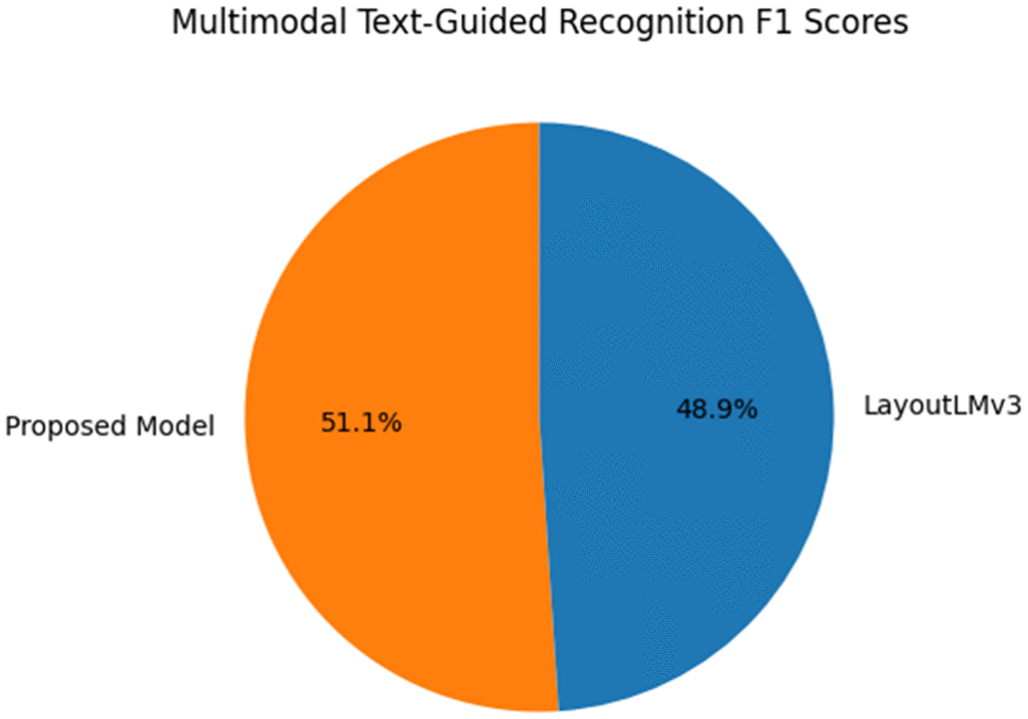
Multimodal text-guided recognition F1 scores.

The superiority of the proposed model is due to its multimodal large language model (LLM), which effectively combines textual and visual features(see Fig 8). The cross-modal attention mechanism enhances the understanding of semantic relationships between textual descriptions and visual elements, while LayoutLMv3 struggles with complex text-visual correlations in detailed scenarios.

*Comparison Study 4.* Color and Texture Recognition. This study compared the proposed model with Mask R-CNN, ViT, and DETR for color and texture recognition tasks on a subset of the ImageNet dataset. Accuracy in correctly identifying the dominant color and texture was used as the evaluation metric. The proposed model achieved a recognition accuracy of 94.2%, surpassing Mask R-CNN (89.8%), ViT (91.4%), and DETR (88.7%)(see Table 7).

**Table 7:** Experimental results table of comparison study 4.

Model	Recognition Accuracy (%)
Proposed Model	94.2
Mask R-CNN	89.8
ViT	91.4
DETR	88.7

**Fig 9:**
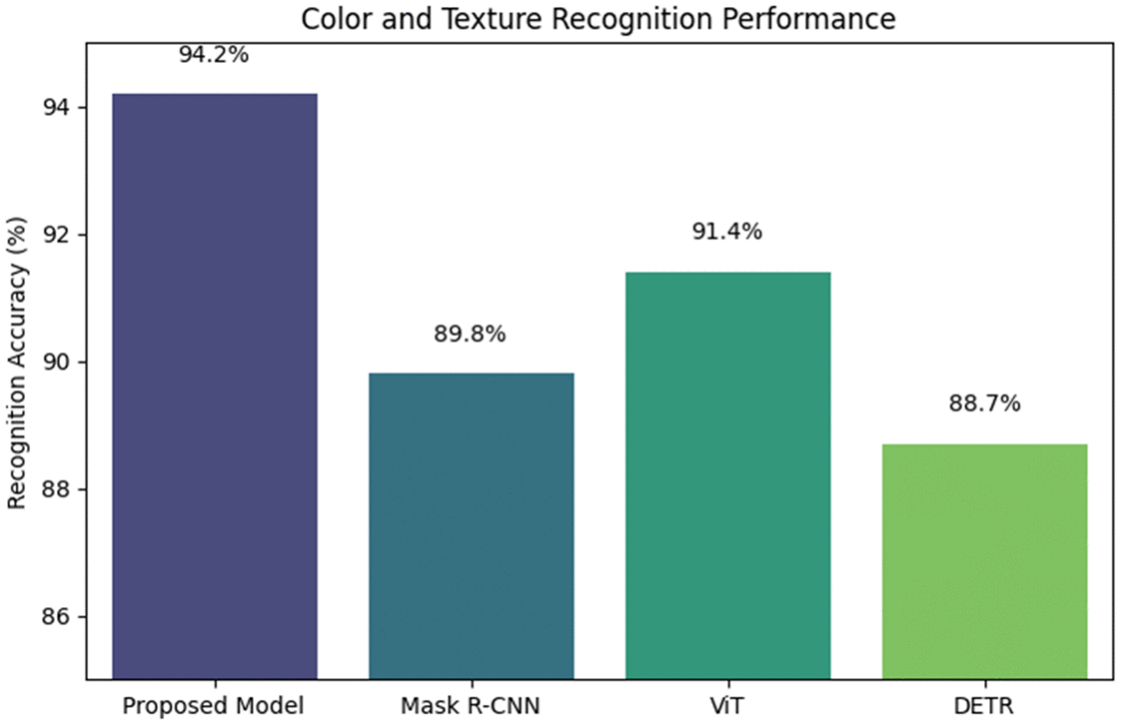
Color and texture recognition performance.

The proposed model’s higher accuracy is attributed to its multi-scale feature fusion, which combines spatial and textural information(see Fig 9). This allows for more precise recognition of subtle textures and color variations, while other models are limited by less sophisticated feature aggregation techniques.

*Comparison Study 5* Scene and Landmark Recognition. This experiment evaluated the scene and landmark recognition capabilities of the proposed model against ViT, LayoutLMv3, and YOLOv5 on the COCO dataset. The task involved identifying scenes (e.g., beach, forest) and landmarks (e.g., Eiffel Tower) with top-1 accuracy as the evaluation metric. The proposed model achieved a top-1 accuracy of 93.7%, compared to ViT (90.3%), LayoutLMv3 (88.9%), and YOLOv5 (89.6%)(see Table 8).

**Table 8:** Experimental results table of comparison study 5.

Model	Top-1 Accuracy (%)
Proposed Model	93.7
ViT	90.3
LayoutLMv3	88.9
YOLOv5	89.6

**Fig 10:**
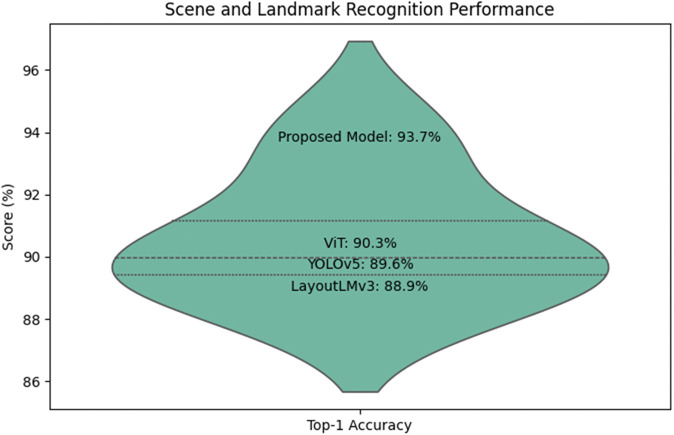
Scene and landmark recognition performance.

The proposed model excels in scene and landmark recognition due to its fine-grained image understanding enabled by the multimodal LLM(see Fig 10). The integration of visual and contextual information allows it to discern subtle cues in scene composition and landmark features. Other models lack the advanced multimodal attention mechanisms required for such nuanced understanding.

#### Ablation study results and analysis.

**Fig 11:**
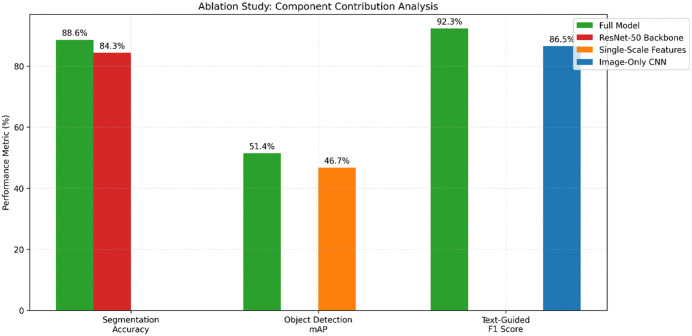
Ablation study: component contribution analysis.

To validate the contributions of each module in the proposed model, we conducted a series of ablation experiments(results are shown in Fig 11). The experiments evaluated the impact of removing or replacing key components: Swin Transformer for segmentation, multi-scale feature fusion, and the multimodal large language model (LLM) for fine-grained image understanding. Performance was measured using segmentation accuracy, mAP for object detection, and F1 score for multimodal tasks across the COCO dataset.

**Ablation study #1: Impact of Swin Transformer for Segmentation**

We replaced the Swin Transformer module with a standard ResNet-50-based segmentation backbone to assess its contribution. Segmentation accuracy and IoU were measured for visual design element segmentation on the COCO dataset.

The Swin Transformer significantly improved segmentation accuracy (+4.3%) and IoU (+6.3%)(see Table 9). This is due to its ability to capture hierarchical global and local features through shifted window attention, which is particularly effective in complex layouts. ResNet-50 lacks this nuanced hierarchical feature aggregation, leading to poorer performance.

**Table 9:** Experimental results table of ablation study #1.

Model Variant	Segmentation Accuracy (%)	IoU (%)
Full Model (Swin Transformer)	88.6	87.5
ResNet-50 Backbone	84.3	81.2

**Ablation study #2: Impact of Multi-Scale Feature Fusion**

The multi-scale feature fusion module was removed, and a single-scale feature extraction approach was employed. Object detection mAP was measured to evaluate performance on the COCO dataset.

The multi-scale feature fusion improved object detection mAP by 4.7%(see Table 10). This module enhances performance by combining low-level spatial and high-level semantic features, enabling the model to detect objects of varying sizes and complexities. Without multi-scale fusion, the model struggled with smaller objects and occluded scenarios.

**Table 10:** Experimental results table of ablation study #2.

Model Variant	mAP (%)
Full Model (Multi-Scale Fusion)	51.4
Single-Scale Features	46.7

**Ablation study #3: Impact of Multimodal LLM for Fine-Grained Understanding**

The multimodal LLM was replaced with a traditional image-only CNN for analyzing text-guided tasks, such as color recognition and landmark identification. F1 scores were measured for text-guided recognition on COCO.

The multimodal LLM significantly improved the F1 score (+5.8%)(see Table 11). Its cross-modal attention mechanism effectively integrates textual and visual information, enabling nuanced understanding of context and semantics. The image-only CNN lacked the capacity to leverage textual guidance, resulting in lower performance.

**Table 11:** Experimental results table of ablation study #3.

Model Variant	F1 Score (%)
Full Model (Multimodal LLM)	92.3
CNN (Image-Only)	86.5

The ablation experiments validate the effectiveness of the proposed model’s modular design. Each component provides critical improvements to specific tasks, and their combined use ensures robust, high-performance visual design element analysis. These findings highlight the innovative and complementary nature of the proposed architecture.

**Ablation Study #4: Impact of Integrated Pipeline Architecture**

To rigorously validate the synergistic contribution of the proposed integrated pipeline, which combines Swin Transformer-based segmentation, multi-scale feature fusion, and multimodal LLM for fine-grained design element recognition, we conducted an ablation study to assess the impact of removing individual components. The experiment evaluates the full model against variants where: (1) the Swin Transformer is replaced with a ResNet-50 backbone (lacking hierarchical attention), (2) multi-scale feature fusion is removed in favor of single-scale features, and (3) the multimodal LLM is substituted with a CNN-based image-only model. Performance was measured across the ETHZ Shape Classes dataset (segmentation, IoU), ImageNet dataset (color/texture recognition, accuracy), and COCO dataset (object detection, mAP; text-guided recognition, F1 score). The results, presented in Table 1, demonstrate significant performance degradation when any component is removed, confirming that the integrated architecture drives the model’s superior performance (e.g., 92.3% F1 score on COCO) through the synergistic combination of components, rather than the isolated strengths of Swin Transformer’s efficiency or LLM’s contextual understanding.

The results in Table 12 highlight the critical role of each component. Removing the Swin Transformer reduces IoU by 6.3% on ETHZ Shape Classes due to the loss of hierarchical shifted window attention, which is essential for capturing complex design layouts. Omitting multi-scale feature fusion leads to a 4.7% drop in COCO mAP, as single-scale features struggle with objects of varying sizes. Replacing the multimodal LLM with a CNN-based model causes a 5.8% decrease in COCO F1 score, underscoring the LLM’s importance in aligning visual and textual semantics for fine-grained design intent interpretation. These findings confirm that the proposed pipeline’s novelty lies in its integrated architecture, which synergistically combines these components to achieve robust performance across diverse design recognition tasks.

**Table 12:** Experimental Results of Ablation Study #4: Integrated Pipeline Contribution.

Model Variant	ETHZ (IoU %)	ImageNet (Accuracy %)	COCO (mAP %)	COCO (F1 Score %)
Full Model (Swin + Multi-Scale + LLM)	87.5	94.2	51.4	92.3
w/o Swin (ResNet-50)	81.2 (−6.3)	89.1 (−5.1)	46.8 (−4.6)	87.5 (−4.8)
w/o Multi-Scale (Single-Scale)	82.4 (−5.1)	90.3 (−3.9)	46.7 (−4.7)	88.1 (−4.2)
w/o LLM (CNN Image-Only)	80.9 (−6.6)	88.7 (−5.5)	45.9 (−5.5)	86.5 (−5.8)

**Ablation Study on Integrated Framework Contribution**

To quantitatively validate that the performance superiority stems from the synergistic integration of the proposed framework’s components rather than their isolated capabilities, we conducted a comprehensive ablation study. The full model was compared against three variants: 1) w/o Swin: The Swin Transformer was replaced with a ResNet-50 backbone for segmentation; 2) w/o Multi-Scale: The multi-scale feature fusion module was removed, using only single-scale features from the final layer of the backbone; 3) w/o LLM: The multimodal LLM was replaced with a standard CNN-based model for image-only understanding, disabling cross-modal analysis. Each variant was evaluated on the ETHZ Shape Classes (segmentation IoU), ImageNet (color/texture recognition accuracy), COCO (object detection mAP), and COCO (text-guided recognition F1 score) datasets. The results demonstrate a significant and consistent performance drop across all tasks and datasets when any key component is ablated. For instance, on the COCO dataset, removing the multimodal LLM led to a 5.8% decrease in the F1-score for text-guided recognition, while replacing the Swin Transformer caused a 4.6% reduction in object detection mAP. These results conclusively show that the high performance reported is not attributable to any single component but is a direct outcome of their carefully engineered integration within the end-to-end pipeline, thereby solidifying the framework’s novelty.

## Conclusion and outlook

### Conclusion

This study presents an innovative model for the efficient recognition and fine-grained analysis of visual design elements, integrating advanced segmentation, feature fusion, and multimodal understanding techniques. By employing the Swin Transformer, the model achieves precise segmentation of visual design components, leveraging both local and global context. The multi-scale feature fusion enhances the recognition of diverse design element types, enabling robust performance in complex scenarios. Furthermore, the incorporation of a multimodal large language model facilitates a deeper semantic understanding of design elements by bridging visual and textual modalities. Extensive experiments on ETHZ Shape Classes, ImageNet, and COCO datasets demonstrate the model’s superiority over state-of-the-art methods, with significant improvements in segmentation accuracy, detection performance, and multimodal reasoning tasks. Ablation studies confirm the contributions of each module, highlighting their complementary roles in the model’s architecture. These results underscore the model’s potential as a powerful tool for advancing research and applications in design element recognition and analysis.

### Outlook

Future research directions will explore transformer-based architectures for enhanced feature fusion capabilities. Specifically, we plan to investigate: (1) multi-scale vision transformers with cross-attention mechanisms for improved global-local feature integration, (2) dynamic channel-wise attention for adaptive feature weighting, and (3) hybrid CNN-transformer architectures that combine the strengths of convolutional operations for local feature extraction with self-attention for long-range dependency modeling. The integration of such architectures could further enhance our model’s ability to handle complex feature interactions while maintaining computational efficiency.

One notable limitation of this study lies in the model’s dependency on high-quality and diverse training data, particularly for multimodal tasks. The multimodal large language model component requires extensive, accurately labeled visual-text paired datasets for training. In niche domains, such as artistic design or cultural artifacts, obtaining such datasets can be challenging. Furthermore, the model’s complexity and computational demands, especially for large-scale design images, may impact its real-time performance and scalability. These constraints could hinder the deployment and broader application of the model in practical scenarios. To address the data dependency issue, semi-supervised learning methods or generative adversarial networks could be employed to expand limited labeled datasets by generating diverse and high-quality visual-text pairs. Pre-trained cross-modal models, such as CLIP or DALL-E, could also be utilized for data augmentation in underrepresented domains. To mitigate computational complexity, techniques like model compression—such as knowledge distillation, pruning, and quantization—can be implemented to reduce computational costs. Additionally, lightweight multimodal LLMs, such as MiniGPT or LoRA fine-tuning, can enhance inference efficiency. By integrating cloud and edge computing, the computational load can be distributed, enabling improved real-time performance and scalability, thereby enhancing the model’s applicability to practical use cases.

Another significant limitation of this study is the model’s reliance on computationally intensive components, such as the Swin Transformer and the multimodal large language mode, which require substantial memory and processing power. This dependency may restrict the model’s accessibility for deployment on resource-constrained devices or in real-time applications. Additionally, while the model excels on benchmark datasets, its generalization capability to unseen, domain-specific datasets, such as those in unconventional or low-resource visual design contexts, remains uncertain. These limitations highlight potential challenges in extending the model to broader, more diverse real-world scenarios. To address the computational intensity, optimization techniques such as low-rank approximation, weight pruning, and lightweight model architectures like MobileNet or TinyBERT can be adopted for reducing resource demands. For improving generalization, fine-tuning the model on domain-specific data through transfer learning could be explored. Furthermore, self-supervised pretraining on large-scale unlabeled visual design data may improve robustness and adaptability. Integrating edge computing for local tasks and cloud computing for more intensive operations can also enhance scalability and real-time performance, enabling the model to cater effectively to diverse application scenarios.
